# Sustainable sodium alginate hydrogels incorporating banana leaf activated carbon and organo-clay for enhanced dye removal

**DOI:** 10.1038/s41598-025-99343-8

**Published:** 2025-05-09

**Authors:** Esraa G. Arafa, Omayma Fawzy Abdel Gawad, Zienab E. Eldin, Marina Medhat Ibrahim, Shimaa Ahmed Abd-Elghafour, Ali H. M. Osman

**Affiliations:** 1 Department of Chemistry, Faculty of Science, Beni-Suef University, Beni-Suef, 62511 Egypt; 2 Petroleum Chemistry, Faculty of Basic Sciences, King Salman International University, South Saini, Egypt; 3 Materials Science and Nanotechnology Department, Faculty of Postgraduate Studies for Advanced Science (PSAS), Beni-Suef University, Beni-Suef, 62511 Egypt; 4 Chemistry Department, Faculty of Science, Beni-Suef University, Salah Salim St., Beni- Suef, 62514 Egypt

**Keywords:** Actived carbon, Montmorillonite clay, Hydrogels, Dyes, Adsorption isotherm, Kinetics, Environmental sciences, Chemistry, Materials science

## Abstract

New sodium alginate-based hydrogels using activated carbon from banana leaves and organo-modified montmorillonite for water treatment. Activated carbon extracted successfully from banana leaves and montmorillonite clay was surface-modified using cetyltrimethylammonium bromide as a cationic surfactant. Hydrogels were then synthesized using calcium chloride as the cross-linking agent. They were characterized using FTIR, X-ray diffraction, and scanning electronic microscopy. Characterization intimated the incorporation of components successfully. Adsorption performance was determined using pH, adsorbent dosages, initial dye concentration, and contact time. Sodium alginate-based hydrogels demonstrated remarkable efficacy in removing MB and EBT dyes from synthetic solutions, achieving removal efficiencies of up to 80.3% and 84.9% respectively within 90 min at pH 7. The adsorption process corresponded better to the Freundlich isotherm model. The kinetics of EBT dye removal were described by a pseudo-second-order model. Meanwhile, the kinetics of the removal of MB dyes were described by both pseudo-first order and intraparticle diffusion models. We conducted MTT assays to determine the cytotoxicity of our blends. This showed a dose-dependent drop in viability. Sodium alginate-based hydrogels made the cells least cytotoxic. The developed hydrogels can be used as safe and effective agents for water treatment, as indicated by the results.

## Introduction

Water treatment benefits greatly from the use of bio-waste. Waste products like activated carbon or biosolids can be used to remove heavy metals and other impurities from water sources. By using a sustainable strategy, waste disposal is decreased and water treatment systems’ overall efficacy and cost-effectiveness are improved. These biomasses biodegrade, producing substantial CO_2_ emissions that play a major role in global warming. A portion of these biomasses are utilized to produce thermal and electrical energy^1,2^. Energy production by burning these biomasses also produces CO_2_. By using the carbon in biomasses to manufacture advanced carbon composites, farmers can earn more money while reducing their carbon footprint. Furthermore, by doing this, valuable petroleum-based compounds that would otherwise be used as precursors for carbon-based products are saved. Creation of heteroatom-doped high surface area activated carbon and its derivatives from a variety of biomasses, including sawdust^3,4^, wheat straw^5^, cornstalk^6,7^, potato waste^8^, bamboo chips^9^, pomelo peel^10^, cotton^11^, peanut meal^12^, hemp stem^13^, coffee ground^14^, banana peel^15^ and orange peel^16-19^ have been reported in the literature. These activated carbons find applications as adsorbents for toxic chemicals and heavy metal ions^4,20-25^, electrodes in batteries and supercapacitors^8,18,26,27^, catalyst supports^28^, and catalysts for oxygen reduction reactions^29^. Since banana plants can produce up to 80% of their output as waste, agricultural waste from bananas, particularly the leaves, is a promising source of adsorbents. For every tonne of harvested banana fruit, around 480 kg of leaves are generated^30^. Banana leaf-based adsorbents are further differentiated by their exceptional stability, excellent efficacy over a wide pH range, safety, ease of use, and environmental friendliness, they are also insoluble in most solvents^31-33^. Because sodium alginate (NaAlg) is a biodegradable, renewable, affordable, and environmentally safe substance, it is obtained from algae (about 21%) and applied extensively to create composite beads that remove impurities from water^34^ and is highly soluble in water^35^. When divalent cations, like Ca^2+^, are added to NaAlg aqueous solutions, stable coagulation occurs at room temperature^36^. Numerous hydroxyl (− OH) and carboxyl (− COOH) groups, which are active adsorption sites utilized to remove organic pollutants such as dyes, pesticides, and antibiotics, are present in the molecular structure of NaAlg^37^. But NaAlg’s stability is a problem, so before using it in aqueous systems for the adsorptive removal of contaminants, it must be modified by physicochemical techniques, such as adding inorganic materials into the NaAlg matrix to prepare composite beads and improve their surface-specific areas and stabilities^38-45^. The incorporation of sodium alginate into the hydrogel appears to enhance the structural integrity of the activated carbon. When activated carbon is embedded within the alginate matrix, the hydrogel network helps prevent the dispersion or aggregation of the carbon particles, thereby maintaining the porosity and surface area of BLAC. Moreover, sodium alginate’s ability to form a stable cross-linked network helps to protect the surface functional groups of BLAC, reducing potential deactivation due to external factors (e.g., mechanical stress, chemical interactions). Montmorillonite (MMT) is a clay mineral classified as a dioctahedral smectite, characterized by its 2:1 layer phyllosilicate structure, consisting of two silica tetrahedral sheets sandwiching one alumina octahedral sheet. Owing to its large specific surface area, high cation exchange capacity, significant adsorption properties, and high porosity, MMT is a versatile material suitable for a wide range of applications. In drug delivery systems, MMT serves as a pharmaceutical excipient, leveraging its strong adsorption capacity to enhance drug entrapment and sustain drug release. It is extensively utilized in various environmental applications, such as soil remediation and water purification, due to its exceptional adsorption capacity and ion-exchange properties^45^.The material montmorillonite (MMT) clay is frequently utilized and affordable for eliminating impurities from aqueous systems^46^. Mt is frequently added in the form of composite beads to improve the durability and functional capabilities of biodegradable polymers like alginate and chitosan^44,47,48^. Additionally, atomic models for Mt, like SAz-2, indicate that − OH forms hydrogen bonds on the surfaces of aluminosilicates, significantly improving the pore structures of alginate aerogels with multiple hydrogen bonding networks. Sodium alginate (NaAlg) plays a critical role as the matrix in the composite hydrogel (NaAlg/BLAC/OMMT). As a natural polysaccharide, sodium alginate serves as an excellent biopolymer for forming hydrogels due to its ability to form cross-linked networks in the presence of divalent cations, such as calcium ions (Ca²⁺), which we used as a cross-linking agent. The NaAlg matrix provides a stable structure for the incorporation of both activated carbon (BLAC) and organo-modified montmorillonite (OMMT), ensuring that these components remain evenly distributed within the hydrogel. The hydroxyl and carboxyl groups of sodium alginate interact with the functional groups on the surface of BLAC, improving the adsorption of dye molecules from aqueous solutions. The NaAlg/BLAC/OMMT hydrogel’s performance in dye removal (e.g., MB and EBT dyes) is attributed to the synergistic effect of sodium alginate, which helps in stabilizing BLAC within the hydrogel, thus enabling efficient dye adsorption. Additionally, the NaAlg network may contribute to the increased contact time between the dye molecules and the adsorbent surface, further improving adsorption efficiency.

In this study, we aim to extract activated carbon from banana leaves as a sustainable source to be used as an active substance in water treatment and also to recycle banana farm waste. Montmorillonite MMT was modified by Cetyltrimethylammonium bromide as a surfactant. Combining all three substances sodium alginate, activated carbon, and modified MMT to prepare hydrogels (NaAlg/ BLAC/OMMT) was successfully done using CaCl_2_ with different clay ratios. The obtained materials were subjected to various analytical techniques such as FTIR, XRD, and SEM to confirm the hydrogel formation. An adsorption study was carried out as a function of pH, the effect of adsorbent dosage, initial dye concentration, and contact time. Cytotoxicity Assessment of the prepared materials was carried out using MTT assay. The results showed that combining sodium alginate, activated carbon, and modified MMT enhances their adsorption capacity.

## Materials and methods

### Materials

The banana leaves were collected from Nasser farm, Beni-Suef district, Egypt. Sodium alginate (Na-Alg; (C_6_H_9_NaO_7_)n, MW = 216.12 g/mol, Assay ≥ 99.0%), Techno Pharmchem, India. Montmorillonite MMT (Na^+^-MMT) with a cation exchange capacity (CEC) of 0.90 meq/g under the trade name minral colloid PB, was purchased Southern clay products Inc. Cetyltrimethylammonium bromide as a surfactant (CTAB, from Merck, Darmstadt, Germany) suitable intercalating agents for MMT type clays because of their long chain molecules with hydrophilic quaternary amine salt heads and organophilic nonpolar alkyl tails. They are stable, cheap and are widely available.

Organic dyes such as Eriochrome black T (EBT; C_20_H_12_N_3_NaO_7_S, assay = 99%) and Methylene blue (MB; C_16_H_18_N_3_SCl, assay = 99%), PIOCHEM, Egypt. Calcium Chloride anhydrous (CaCl_2_) with assay = 96%, Sodium Hydroxide (NaOH) with assay = 98%, and Hydrochloric Acid (HCl) with minimum assay 35 _38%, PIOCHEM, Egypt.

## Methods

### Preparation of activated carbon (AC) from banana leaves

The method described by Srinivasakannan and Bakar involved using Banana leaves as a starting material to produce activated carbon^49^. To begin, the Banana leaves were dried in an oven at 70˚C overnight. Next, the dried leaves were soaked in a 2% NaOH solution overnight. After soaking, the samples were subjected to semicarbonization in a muffle furnace at 200˚C for 4 h. The semicarbonized material was then dried and cooled to room temperature. Subsequently, the dried material underwent further heating in a furnace at 500˚C for 2 h to activate it. The resulting activated material, known as BLAC, was washed multiple times with distilled water until all traces of sodium hydroxide were removed, continuing until the wash solution became neutral. Finally, the product was dried in a hot air oven for 5 h at 105˚C.

### Preparation of modified clay MMT (OMMT)

Unmodified MMT powder (500.0 mg) was dispersed in 400 mL of hot H_2_O. Different ratio of CTAB (500, 250, and 165 mg) was dissolved in 100 mL hot H_2_O and slowly added into the montmorillonite dispersion at 70 ^o^C. Following this addition, the suspension was sonicated overnight at 70 °C using an ultrasonic bath and allowed to equilibrate for 12 h at room temperature. The resulting precipitate was filtered and washed multiple times with hot deionized water until bromide ions were no longer detected using an AgNO_3_ solution. The obtained organo-clay was dried in a vacuum oven at 70 ^o^C for 12 h and ground in a mortar to obtain fine powder, which was denoted as CTAB-MMT, Table 1.

**Table 1:** OMMT samples.

Sample cods	MMT	CTAB
CTAB-MMT 1:1 (OMMT_1_)	500 mg	500 mg
CTAB-MMT 0.5:1 (OMMT_2_)	500 mg	250 mg
CTAB-MMT 0.33:1 (OMMT_3_)	500 mg	165 mg

### Preparation of the sodium alginate - activated carbon/OMMT hydrogels (NaAlg/ BLAC/OMMT)

One gram of NaAlg (2%) was dispersed in 50 ml of H_2_O at 45 °C, and mechanically stirred for 30 min. Then, 0.25 g of modified clay OMMT_1_, and 0.25 g of BLAC were added to 20 ml of H_2_O. The mixture was stirred using a magnetic stirrer for 15 min, followed by sonication for 30 min. The two prepared solutions, were then mixed together and stirred for one hour. Additionally, one gram of CaCl_2_ (1%) wt/v as the cross linker was dispersed in 100 ml of H_2_O to prepare the cross linker solution. This solution was gradually dropped to the mixture until gelation occurred. The prepared hydrogel was left to dry at 40 °C for 24 h. Hydrogels with other modified clays (OMMT_2_, and OMMT_3_) were prepared via the same method.

### Characterization of the prepared materials

The materials that were prepared underwent characterization using various techniques. Fourier transform infrared spectroscopy (FTIR) was performed on a Bruker-Vertex 70 instrument using the KBr pellet technique, covering a spectral range of 400–4000 cm⁻¹ to analyze functional group vibrations. X-ray diffraction (XRD) analysis was conducted on a PANalytical Empyrean instrument (Sweden) with Cu-Kα radiation (λ = 0.154 nm) operating at 35 mA and 40 kV. The scanning range was set from 5° to 80° (2θ) at a rate of 8° min⁻¹ to assess the crystallinity of the materials. Surface morphology was examined using a Zeiss Sigma 500 VP Field Emission Scanning Electron Microscope (FESEM, Germany), providing detailed insights into surface characteristics. Additionally, X-ray photoelectron spectroscopy (XPS) was performed on a K-ALPHA instrument (Thermo Fisher Scientific, USA) with monochromatic Al Kα radiation (10–1350 eV), a spot size of 400 μm, and a pressure of 10⁻⁹ mbar. The full spectrum was acquired at a pass energy of 200 eV, while the narrow spectrum was recorded at 50 eV.

### Hydrogels swelling measurements

The swelling behavior of the hydrogels was investigated by immersing 0.1 g of hydrogel (W_0_) in 25 mL of distilled water at room temperature for various time intervals ranging from 15 to 300 min. At regular intervals, the swelled hydrogel was gradually taken out of the aqueous solution, wiped with filter paper to get rid of extra surface water, and weighed (Ws). The percentage of swelling degree was determined using the following Eq. 1:

1 $$\:Swelling\:degree\%=\left[\frac{Ws-{W}_{0}}{{W}_{0}}\right]\times\:100$$

Where Ws and W_0_ represent the weight of swollen and the dry hydrogel, respectively.

### Dye adsorption measurements

High-quality Eriochrome black T (EBT) and Methylene blue (MB) were used to create a stock solution with a concentration of 1000 mg/L. All adsorption experiments were conducted using the batch method. Specifically, 20 mg of NaAlg/BLAC/OMMT_3_ was dispersed in separate 20 mL solutions containing 10 mg/L of the respective dyes. The adsorption process took place on a shaker at room temperature with a fixed rotation rate of 140 rpm. After 24 h, the adsorbent was magnetically separated from the dye solutions, and the residual dye concentration was quantified via UV-visible spectrophotometry (Shimadzu UV-1800) at λmax = 490 nm for EBT and 663 nm for MB.

First of all, the impact of pH on the adsorption capacity of the adsorbents for EBT and MB was investigated. The initial pH of a 10 mg/L dye solution was adjusted within the range of 2–8 by adding either 0.1 M HCl or NaOH solutions. To examine the adsorption kinetics, 20 mg of the adsorbents were dispersed in 20 mL of a 10 mg/L dye solution at pH 7. At regular time intervals, the concentrations of EBT and MB were measured. For the adsorption isotherms, the initial concentration of dyes was adjusted within the range of 5-100 mg/L, at a pH of 7, and left for 24 h. The equilibrium adsorption capacities were determined using Eq. 2, which involved calculating the difference between the initial concentration and the final concentration at equilibrium time. The percentage removal of dye was calculated Eq. 3.

2 $$\:\left[{q}_{e=}\frac{\left({C}_{0-}\:{C}_{e}\right)V}{W}\right]$$

3 $$\:Dye\:removal\:\%=\:\frac{{C}_{0}-{C}_{e}}{{C}_{0}}\:\times\:100\:$$

where, C_o_ and C_e_ (mg/L) represent the initial and final concentrations of MB and EBT dyes in the solutions of adsorption. Additionally, q_e_ (mg/g) signifies the adsorption equilibrium of adsorbents for dyes. The dye adsorption trials were repeated three times, and the resultant data was averaged, and reported as the mean value with a ± 5 range.

### Regeneration (desorption) studies

The desorption investigations of methylene blue (MB) and Eriochrome black T (EBT) loaded NaAlg/BLAC/OMMT_3_ hydrogel aimed to investigate the potential for reusing the NaAlg/BLAC/OMMT_3_ hydrogel adsorbent. The MB or EBT loaded NaAlg/BLAC/OMMT_3_ hydrogel was placed in a glass container with 50 mL of 0.1 M HCl solution and agitated at room temperature for 24 h. Following this immersion period, the hydrogel was utilized once more for dye adsorption. This adsorption-desorption process was repeated five times, employing a fresh dye solution each time under identical experimental conditions. The adsorption capacity of the NaAlg/BLAC/OMMT_3_ hydrogel was determined after each of these cycles^50^.

### Cytotoxicity assessment

#### Cell lines

The cell lines utilized in this paper, was obtained from the Tissue Culture unit of the Holding Company for Biological Products and Vaccines (VACSERA) in Giza, Egypt. Vero cells, a cell line originating from African green monkey kidney tissue and WRL-68 (normal liver cell line) were cultured in RPMI-1640 medium supplemented with 10% fetal bovine serum (FBS), 2 mM glutamine, and antibiotics (100 units/mL penicillin and 100 µg/mL streptomycin). The cell cultures were maintained at 37 °C in a humidified atmosphere containing 5% CO_2_.

#### MTT assay

The MTT assay was employed to assess the cytotoxicity of NaAlg/BLAC/OMMT hydrogel and hydrogel after adsorption MB and EBT on Vero cells. This method quantifies cell viability by measuring the transformation of MTT into colored formazan by metabolically active cells. The experimental procedure involved seeding Vero cells (1 × 10^4 cells/well) in 96-well plates for 24 h. Following washing and media replacement, the cells were treated with varying concentrations of hydrogel befor and after adsorption (7.8–1000 µg/mL) in triplicate for 24 h. Post-incubation, the cells were washed with PBS and exposed to MTT solution (20 µL, 5 mg/mL) for 4 h. After discarding the culture medium and excess dye, DMSO (100 µL) was added to dissolve the formazan crystals. The mixture was then agitated for 15 min, and the absorbance was measured at 570 nm using a microplate spectrophotometer.

## Results and discussions

### Characterizations

#### FT-IR spectra

**Fig. 1:**
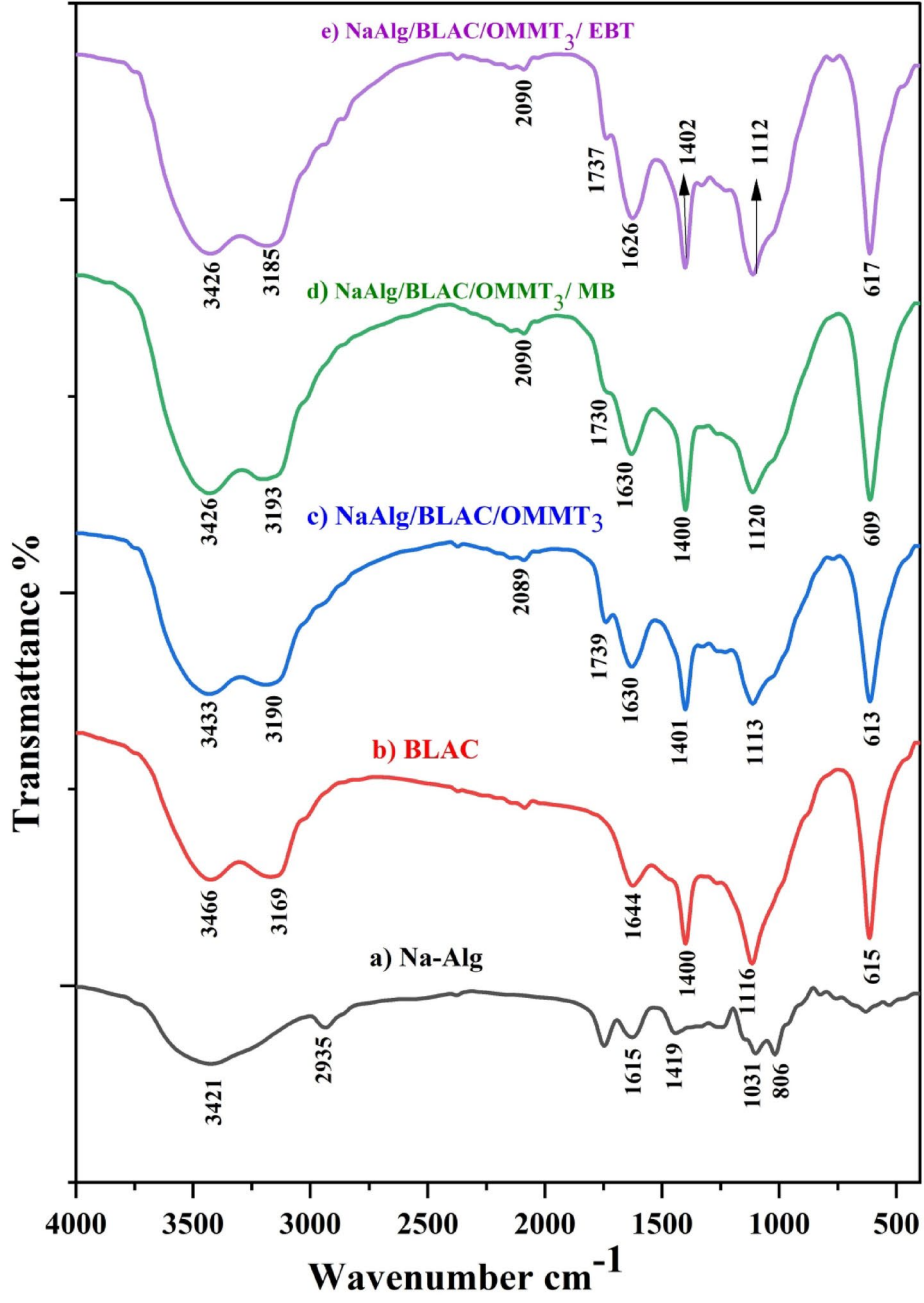
FT-IR spectra of NaAlg (f), BLAC (b), NaAlg/BLAC/OMMT_3_ (c), NaAlg/BLAC/OMMT_3_/ MB (d), and NaAlg/BLAC/OMMT_3_/ EBT (e).

**Fig. 2:**
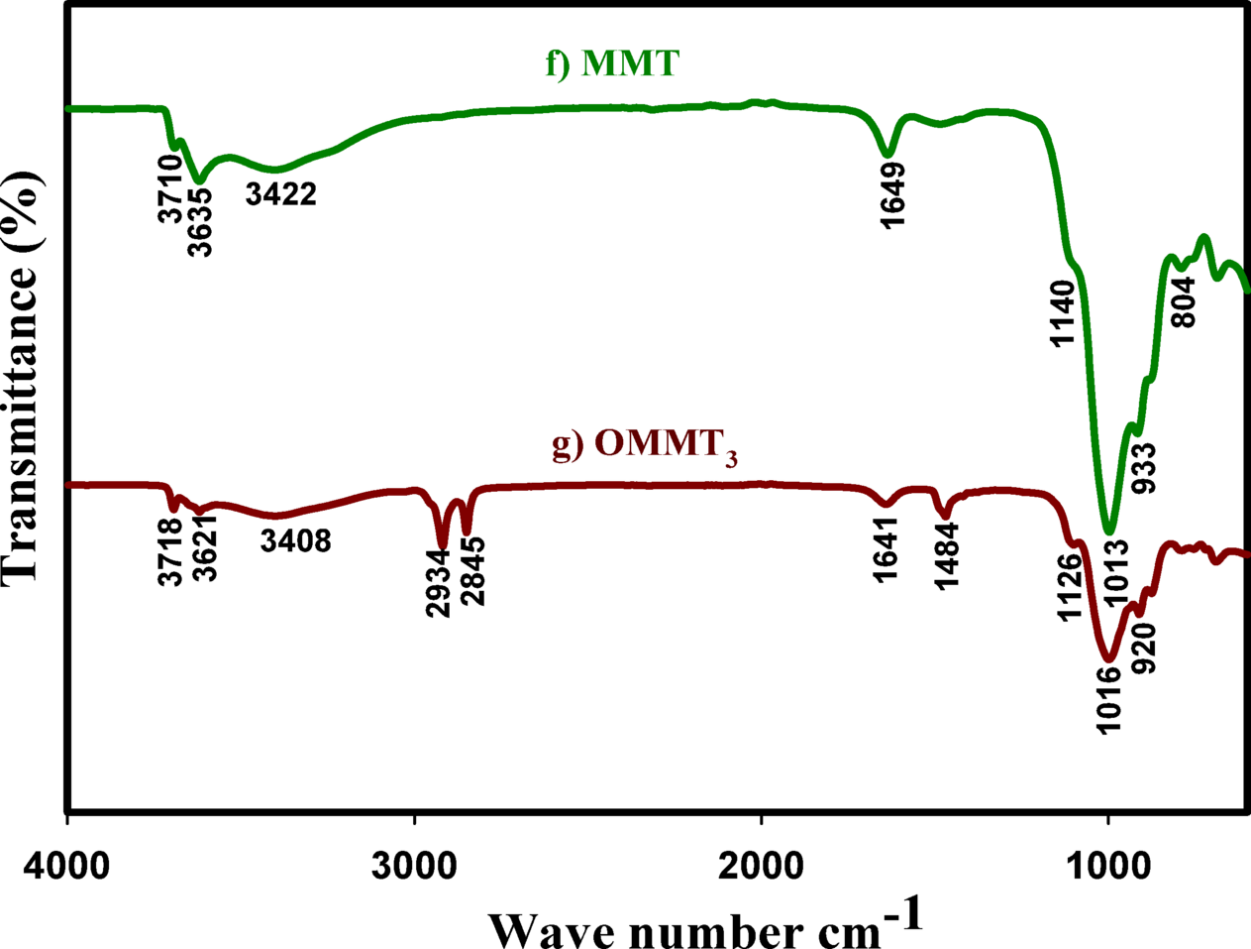
FT-IR spectra of MMT (f), OMMT_3_ (g).

The investigation focused on the alteration of chemical groups as a reliable indicator for the formulation of effective hydrogels organo-clay composites NaAlg with BLAC/OMMT_3_ and the successful loading of the obtained hydrogels organo-clay composites by MB, and EBT dyes (Fig. 1). The FT-IR spectra of NaAlg, MMT, OMMT_3_, BLAC, NaAlg/BLAC/OMMT_3_ loaded with MB, and NaAlg/BLAC/OMMT_3_ loaded with EBT were represented graphically in Fig. 1, and Fig. 2. The Fourier Transform Infrared (FTIR) spectra of NaAlg featured prominent absorption broad band 3421 cm^− 1^ corresponding to the O-H stretching vibration band of its structural-functional groups; the peaks at 1615 cm^− 1^ and 1419 cm^− 1^corresponded to –COO^−^ stretch vibration, respectively. A peak is observed at finger print region around 800–600 cm^− 1^ due to the presence of the Na–O stretching (Fig. 1a)^51,52^.

Also, the pure phase of BLAC showed its characteristic spectrum reflecting the presence of stretching vibrations of C = C, O–H, C-O, bending CH- functional groups, and the band attributed to the C–O–H group rocking vibrations of cellulose at the absorption bands of 1644 cm^− 1^, 3466 cm^− 1^, 1116 cm^− 1^, 1400 cm^− 1^, and 617 cm^− 1^ respectively^31^ (Fig. 1b).

The FTIR spectrum of MMT (Fig. 2f), The unique absorption bands at 3422 cm^− 1^ in MMT are attributed to the –OH stretching band of adsorbed water. The bands at 3635 and 3710 cm^− 1^ are due to –OH band stretch for Al–OH and Si–OH. The shoulders and wideness of the structural –OH band mostly result from the presence of many structural –OH groups in the MMT layer. The overlaid absorption peak at 1649 cm^− 1^ is attributed to –OH bending mode of adsorbed water. The primary peaks observed at 1140 and 1013 cm^− 1^ correspond to the out-of-plane and in-plane stretching vibrations of Si–O bonds in layered silicates, respectively. Peaks at 933, 804, and 790 cm^− 1^ are attributed to AlAlOH, AlFeOH, and AlMgOH bending vibrations, respectively^53-55^. FTIR spectrum of OMMT_3_ (Fig. 2g), shows Multiple absorption bands, including MMT characteristic bands, as well as novel absorption bands at 1484, 2845, and 2934 cm − 1, suggest the existence of long alkyl chains in the clay. This observation implies that CTAB interacts strongly with the MMT layers^56-58^.

The Fourier transform infrared spectrum of hydrogels organo-clay composites NaAlg with BLAC/OMMT_3_ exhibited several complicated bands between NaAlg and BLAC/OMMT_3_, which deviated observably from their positions in the pure phases (Fig. 1c). This sample contains 16.6% of OMMT_3_ and 66.6% of sodium alginate, 16.6% of BLAC and it is easy to observe that the spectrum is a mix of NaAlg, OMMT_3_, and BLAC spectrum.The main detected groups are –OH stretching of BLAC that appeared in overlap position with –OH group of NaAlg (3433 cm^− 1^). A new two strong bands which shifted from 1615 to 1419 cm^− 1^ to 1630 cm^− 1^ and 1401 cm^− 1^ indicates overlap COO^−^ stretching of NaAlg with the C = C stretching vibration band of BLAC. Moreover, the peak corresponding to Si–O stretching (out-of-plane) vibration for layered silicates was also found which shifted from 1016 cm^− 1^ to 1113 cm^− 1^. Additionally, C–O–H group rocking vibrations of cellulose was also identified at 613 cm^− 1^. In brief, all the results stated that NaAlg was effectively composites to NaAlg /BLAC/OMMT_3_^59,60^.

The FT-IR spectrum of NaAlg/BLAC/OMMT_3_ loaded with MB, and NaAlg/BLAC/OMMT_3_ loaded with EBT was presented in comparison with the FT-IR spectrum of hydrogels organo clay composites NaAlg with BLAC/OMMT_3_ (Fig. 1c) without loading and NaAlg/BLAC/OMMT_3_ loaded with MB, and NaAlg/BLAC/OMMT_3_ loaded with EBT (Fig. 1d), and (Fig. 1e) respectively. The spectra of the synthetic hydrogels organo-clay composites after loading reveals prominent bands that exhibit significant changes in their position, indicating the interaction effect of the added MB or EBT fluorescent dye molecules. Table 2 reports that, in comparison, the MB-loaded and EBT-loaded adsorbents showed major band shifts in band intensity. The electrostatic binding between the cationic and anionic dyes and the NaAlg/BLAC/OMMT_3_ hydrogel network may be the cause of the slight variations in absorption peak frequencies. The observed changes in absorbance suggest that dye-binding mechanisms were occurring on the adsorbents’ active sites.

**Table 2:** The FT-IR peak positions of NaAlg/BLAC/OMMT3, NaAlg/BLAC/OMMT3 loaded with MB, and NaAlg/BLAC/OMMT3 loaded with EBT before and after adsorption. Absroption band peak (cm^− 1^) before, and after loaded for O–H, C = O, C = C, COO-, C-O, and C-OH.

Adsorbent	Absroption band peak (cm^-1^)			Functional groups
	Before	After	Difference	
NaAlg/BLAC/OMMT_3_ loaded with MB	3433 3190	3426 3193	7 3	O-H
NaAlg/BLAC/OMMT_3_ loaded with EBT	3433 3190	3426 3185	7 5	O-H
NaAlg/BLAC/OMMT_3_ loaded with MB	1739	1730	9	C = O
NaAlg/BLAC/OMMT_3_ loaded with EBT	1739	1737	2	C = O
NaAlg/BLAC/OMMT_3_ loaded with MB	1630	1630	-	C = C, COO^-^
NaAlg/BLAC/OMMT_3_ loaded with EBT	1630	1626	4	C = C, COO
NaAlg/BLAC/OMMT_3_ loaded with MB	1401	1400	1	COO^-^
NaAlg/BLAC/OMMT_3_ loaded with EBT	1401	1402	1	COO^-^
NaAlg/BLAC/OMMT_3_ loaded with MB	1113	1120	7	C-O
NaAlg/BLAC/OMMT_3_ loaded with EBT	1113	1112	1	C-O
NaAlg/BLAC/OMMT_3_ loaded with MB	613	609	4	C-OH
NaAlg/BLAC/OMMT_3_ loaded with EBT	613	617	4	C-OH

### X-ray diffraction

**Fig. 3:**
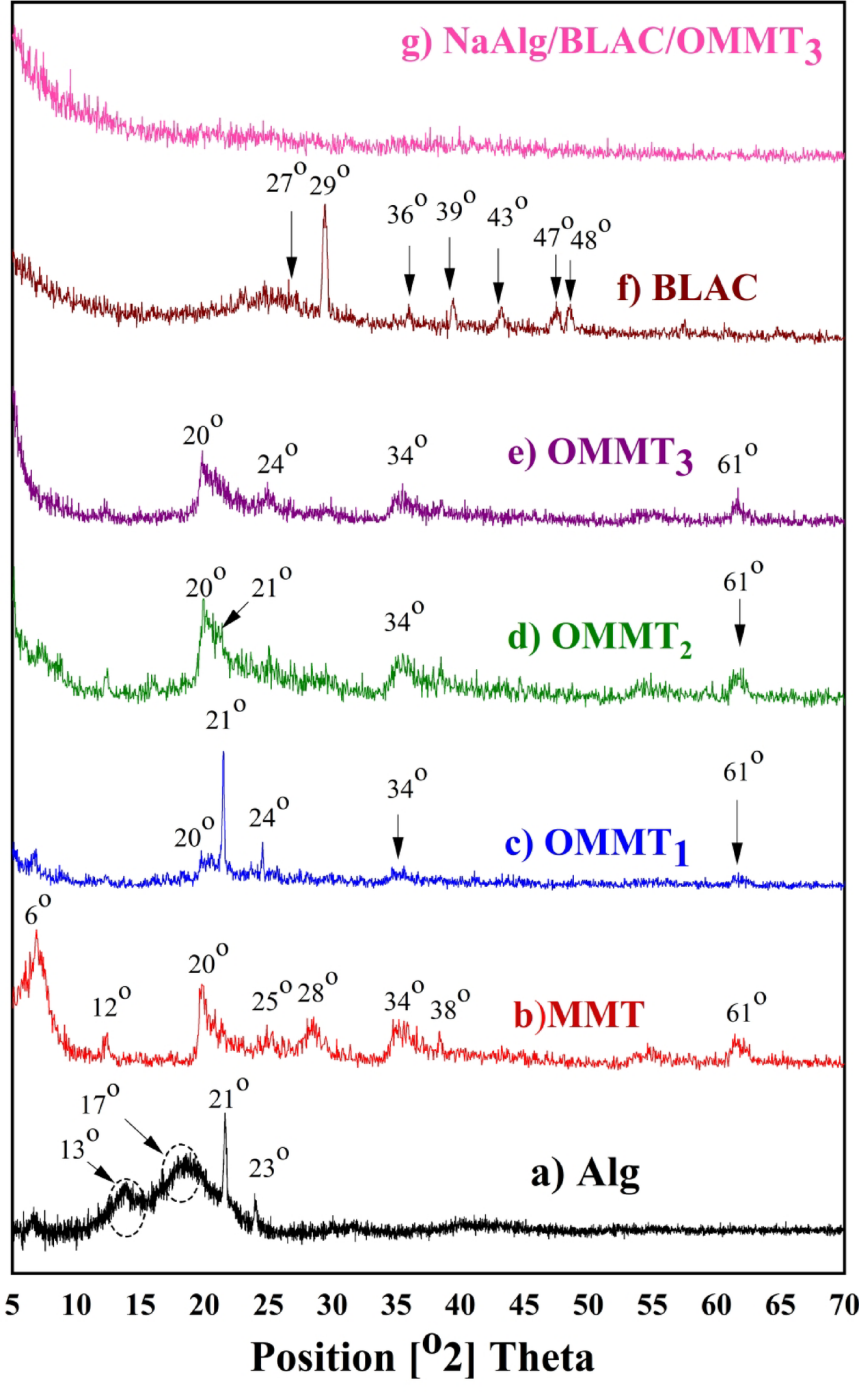
XRD patterns of NaAlg (**a**), MMT (**b**), OMMT_1_ (**c**), OMMT_2_ (**d**) OMMT_3_ (**d**), BLAC (**f**), NaAlg/BLAC/OMMT_3_ (**g**).

The most efficient technique for establishing that surfactants are loaded into the galleries of the resultant organoclays is XRD analysis. Figure 3 illustrates the XRD patterns of NaAlg (a), MMT (b), OMMT_1_ (c), OMMT_2_ (d), OMMT_3_ (e), BLAC (f), and NaAlg/BLAC/OMMT_3_ (g). The detected diffraction peak of NaAlg appeared as two sharp crystalline peaks at around 2θ = 21 ^o^ and 23 ^o^, and two broad peaks at around 2θ = 13^o^ and 17 ^o^ indicating the presence of a hydrated crystalline structure^61,62^ (Fig. 3a).

In (Fig. 3b), a typical diffraction peak of MMT is observed at 19.8^o^ (100)^54,58^. Additional peaks are identified at 6 ^o^, 12 ^o^, 20 ^o^,34 ^o^, 38 ^o^,61^o^ matching with the (003), (006), (009), (105), and (300) mirror planes respectively, according to the JCPDS card (NO. 00-046-1045)^63-65^. After intercalation with the CTAB Fig. 3c and d, and 3e this peak moves to high angle (20.1◦). This shows that the intercalated MMT with CTAB alteration successfully formed^66,67^. Compared the interlayer spacing of the OMMT_3_ (Fig. 3e), to that of OMMT_1_ (d-spacing = 4.13 Å), and OMMT_2_ (d-spacing = 4.41 Å) which had expanded to the highest interlayer spacing value (4.46 Å).

The BLAC sample that was used had a highly crystalline appearance with peaks at 27^o^, 36^o^, 39^o^, 43^o^, 47^o^, and 48^o^ in addition to the intense peak at 29 are attributed to the presence of native cellulose as the prime component, which may be crystalline or amorphous (Fig. 3f)^68^. The XRD patterns at 2θ = 27° (002), and 43° (101) matched well with the reported JCPDS card number (75-2078)^69^.

As shown in Fig. 3g., it is clear that the hydrogels organo-clay composites NaAlg /BLAC/OMMT_3_, The distinctive peaks of BLAC and OMMT_3_ totally disappeared, and the resulting pattern showed the formation of new materials with an amorphous structure, which were represented by low-intensity peaks, NaAlg molecules are able to more readily enter the MMT gallery due to the upgraded MMT’s extended gallery distance. The disordered intercalated structure is most likely indicated by the decrease in intensity of the MMT plane’s diffraction characteristic peak, which suggests that they are associated with the amorphous part of NaAlg^59^.

### SEM analysis

**Fig. 4:**
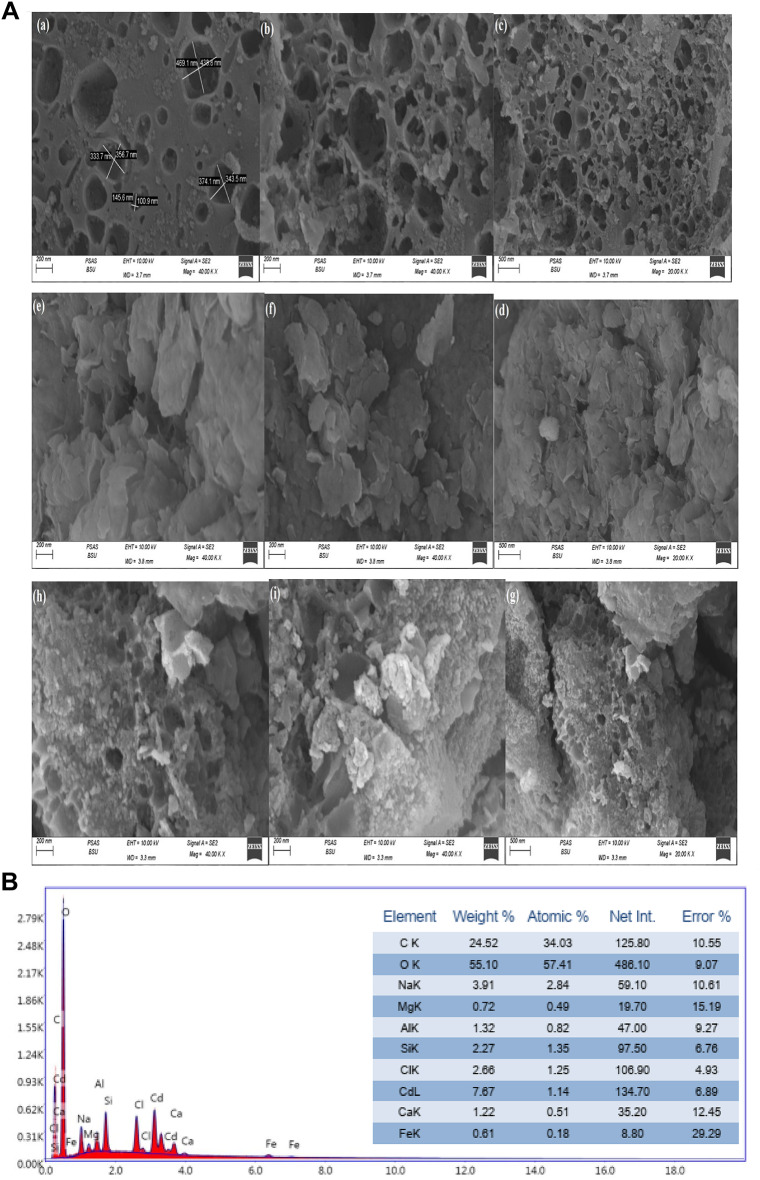
**A**. Showed morphological and internal structures of hydrogel organo-clay composite of NaAlg/BLAC/OMMT_3_; (a, b, and c) the SEM images of pure BLAC, (d, e, and f) OMMT_3_, (g, h, and i) the SEM images of NaAlg/BLAC/OMMT_3_.** B**. The EDS quantitative analysis of NaAlg/BLAC/OMMT_3_ hydrogel.

The morphological properties, as well as the internal structures of the synthetic hydrogel organo-clay composite of NaAlg/BLAC/OMMT_3_, appear in Fig. 4A. In SEM image of BLAC (Fig. 4A; a,b, and c), which exhibited an interconnected network of porous structures spread across the surfaces, containing a thickly layered structure that was highly organized, interconnected, and had a 3D spherical with an average diameter. SEM image of modified MMT is showed in (Fig. 4A; d,e and f) appeared as clay texture consist of multilayered silicate platelets are consisting of parallel alternating clay layers silicate formed from a loosely parallel layers stacked together presented in the form of bulky flakes and aggregated disorderly^51,70^. The close view on the surface of the hydrogel organo-clay composite of NaAlg/BLAC/OMMT_3_ (Fig. 4A; g,h, and i), it can be noticed as the amount of OMMT_3_ clay increases the surface loses uniformity, an increase of the rougher surface exhibited particles sizes with small uneven cavities with micron size on its morphology, and high porosity structure, which explains the elevated composite degree of BLAC with NaAlg^60^. In general, presence of NaAlg in composite induce the higher amorphous nature and less crystalline nature. In short, the change of the surface morphology, establish the existence of a new solid phase in those binary systems due to a decrease in crystalinity and habitus change. This morphology reflected their composition from uneven cavities and sometimes connected with each other forming nearly Honeycomb shape that can give the synthetic hydrogel organo-clay composite with highly porosity surface and more promising water molecules to be absorbed and interacted with hydrophilic groups on dyes. On the other hand, Fig. 4B presents the EDS quantitative analysis of the NaAlg/BLAC/OMMT_3_ hydrogel composite, highlighting its major elements, including C, O, Al, Si, Mg, and Fe, along with minor elements such as Na, Ca, and Cl, attributed to the presence of sodium alginate and the calcium chloride (CaCl₂) used as the cross-linking agent in hydrogel synthesis. Additionally, a trace impurity from montmorillonite (MMT) identified as Cd, was also detected.

### Swelling kinetics

**Fig. 5:**
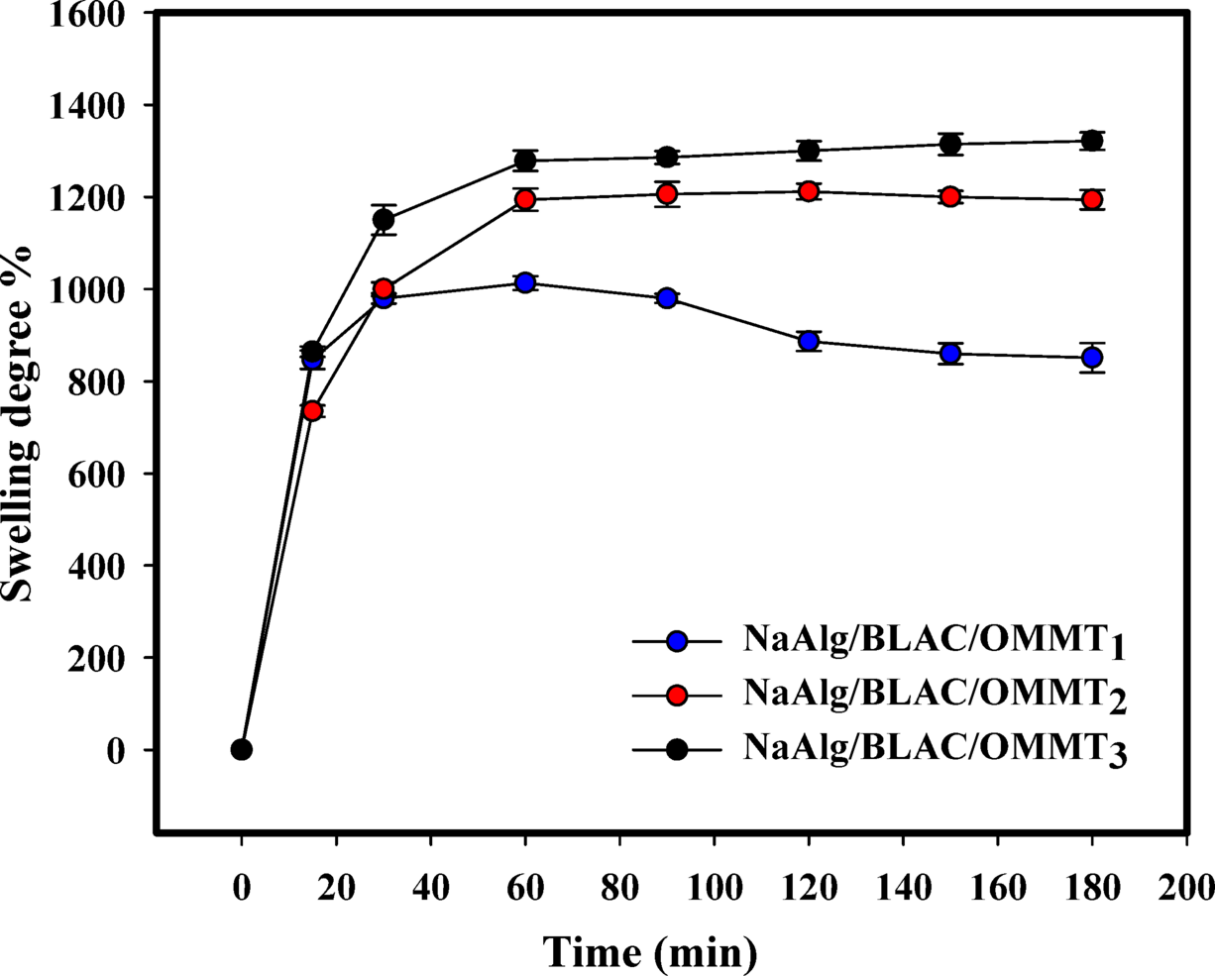
Swelling behavior of hydrogels samples (NaAlg/BLAC/OMMT_1_, NaAlg/BLAC/OMMT_2_, and NaAlg/BLAC/OMMT_3_).

Figure 5 illustrates the equilibrium swelling of the hydrogels that were prepared in aqueous solutions at room temperature (RT) over different time intervals (15–300 min). The swelling degree was examined for hydrogels with different compositions (NaAlg/BLAC/OMMT_1_, NaAlg/BLAC/OMMT_2_, and NaAlg/BLAC/OMMT_3_). Initially, the swelling degree of all hydrogels increased rapidly until reaching the maximum value, known as the equilibrium swelling degree. Subsequently, the rate of increase slowed down considerably until it eventually reached a plateau^71,72^. This can be attributed to the fact that there were initially more gaps and open areas in the hydrogel network structure that facilitated the absorption of water molecules. As time progressed, these spaces diminished, eventually becoming saturated. Noted that the swelling ratios of the hydrogel samples are influenced by the content of OMMT. Among the hydrogels investigated, the NaAlg/BLAC/OMMT_3_ hydrogel exhibited the highest swelling degree. This observation may be attributed to an increase in porosity within the hydrogels. This increase in porosity was confirmed by XRD analysis, which revealed that the NaAlg/BLAC/OMMT_3_ hydrogel possessed the highest basal spacing degree, measuring 4.46 Å. After 90 min, all hydrogels reached their equilibrium swelling.

### Adsorption parameters

#### Effect of pH

The absorption of dye by hydrogels can be explained through the mechanism of absorption transport into swollen hydrogel networks. The hydrogel’s higher water content and porous structure enable the diffusion of solutes throughout the hydrogel structure. When a hydrogel is immersed in an aqueous medium, it begins to absorb water, leading to significant changes in its structure. Whether or not the dye molecules penetrate the hydrogel depends on the interactions established between the dye molecules and hydrogels. It is widely recognized that the pH of the medium has a significant impact on the adsorption performance of hydrogels for dyes. This is because pH influences the binding sites of the hydrogels and the ionization process^73,74^.

**Fig. 6:**
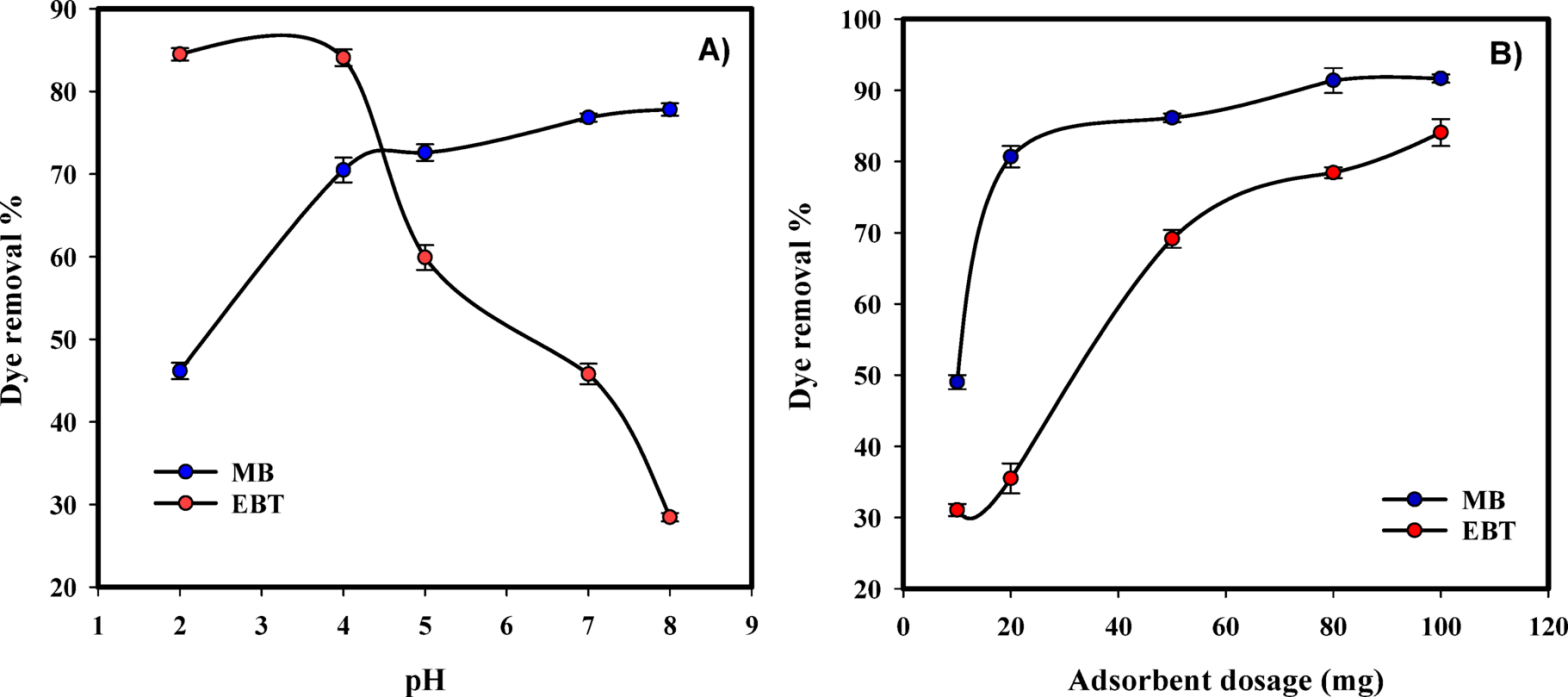
**A**) Effect of pH on MB and EBT adsorption on NaAlg/BLAC/OMMT_3_ hydrogel and.** B**) Effect of adsorbent dosage on MB and EBT adsorption on NaAlg/BLAC/OMMT_3_ hydrogel.

Figure 6a shows the effect of pH variations on the MB and EBT removal from the aqueous solution, for initial dye concentration of 10 mg/ L and 20 mg of NaAlg/BLAC/OMMT_3_ for 90 min. The efficiency of dye removal using NaAlg/BLAC/OMMT_3_ hydrogel was found to be highly dependent on the pH of the solution. In the case of the cationic dye (MB), an increase in pH from 2 to 8 resulted in an increase in the removal percentage by NaAlg/BLAC/OMMT_3_ hydrogel, reaching a maximum removal percentage of approximately 78% at pH 8. On the other hand, for the anionic dye (EBT), a decrease in pH from 8 to 2 led to an increase in the removal percentage by NaAlg/BLAC/OMMT_3_ hydrogel, with a maximum removal percentage of approximately 85% observed at pH 2. At elevated pH levels, the sodium alginate’s COOH groups dissociate, forming COO^−^, which increases the number of ionized groups that are fixed in place. As a result, electrostatic repulsion forces arise between adjacent ionized groups within the polymer networks, causing the polymer chains to expand within the hydrogel structure^75,76^. This phenomenon facilitates the formation of an ionic complex between the MB molecules and the hydrogel networks, resulting in increased removal of MB. Beside, the OMMT surface’s silanol groups undergo increased deprotonation when the adsorption system’s pH increases^77^. Consequently, the number of negatively charged adsorbent sites increases, leading to a higher removal efficiency for MB. Conversely, the enhanced adsorption of EBT at acidic pH levels can be attributed to the presence of an excess of H^+^ ions, which compete with the dye cations for adsorption sites^77^. Activated carbon exhibits basic and acidic characteristics. Carboxylic, lactonic, and phenolic groups are examples of acid functional groups, whereas oxygen-containing species including ketonic, pyronic, chromenic, are examples of basic functional groups, along with the p-electron system found in carbon basal planes^78^. The surface charge of carbon is primarily determined by the pH of the solution containing the adsorbate. A lower solution pH leads to a positive net charge on the carbon surface, thereby enhancing EBT removal, whereas a higher solution pH results in a negative net charge, leading to increased removal of MB^79^. Therefore, the electrostatic attraction between the NaAlg/BLAC/OMMT_3_ hydrogel network and both cationic and anionic dyes is a significant factor contributing to the observable increase in the removal efficiency.

#### Effect of the adsorbent (NaAlg/BLAC/OMMT_3_) dosage

A dosage study holds significant value within adsorption studies as it establishes the adsorbent’s capability to accommodate a specific initial concentration of dye in a solution. Figure 6b shows the effect of NaAlg/BLAC/OMMT_3_ hydrogel dosage variations on the MB and EBT removal from the aqueous solution, for initial dye concentration of 10 mg/L and pH 7 for 90 min. The efficiency of dye removal using NaAlg/BLAC/OMMT_3_ hydrogel was found to be increased with increase the absorbent dosage in the solution. In the case of the cationic dye (MB), an increase in dosage from 10 to 100 mg resulted in an increase in the removal percentage from 49 to 91.60% after 90 min. Also, in the case of the anionic dye (EBT), an increase in dosage from 10 to 100 mg resulted in an increase in the removal percentage from 31 to 84% after 90 min. This occurrence was attributed to the presence of numerous adsorption sites. Dyes would randomly come into contact with and bind to the adsorption sites within the hydrogel. As the number of sites increased, the probability of contact was significantly heightened, resulting in an enhancement in dye removal^80^. These findings align with the surface morphology of the NaAlg/BLAC/OMMT_3_ hydrogel, which exhibits a structured construction characterized by a greater abundance of pores and cavities as adsorption sites^40,81^.

### Adsorption isotherm

To understand how molecules interact with adsorbents and how the molecules distribute between solid and liquid phases during an equilibrium adsorption process, it is necessary to optimize the NaAlg/BLAC/OMMT_3_ hydrogel adsorbent for removing pollutants from the environment^82,83^. In this context, experimental data on the adsorption of MB (methylene blue) and EBT (Eriochrome black T) were utilized to determine the isotherms.

For this aim, two commonly used isotherm models, namely the Langmuir and Freundlich models were utilized. The Langmuir isotherm assumes that the adsorbent’s surface is uniform and approximates the maximum monolayer adsorption capacity. It describes the adsorption process as a single layer of molecules being adsorbed onto the surface of the adsorbent. The Langmuir isotherm equation is often used to calculate the maximum adsorption capacity and the equilibrium constant^84^. On the other hand, the Freundlich isotherm is utilized to characterize the adsorption of multiple layers onto a heterogeneous surface of the adsorbent. It assumes that the active surface sites are distributed exponentially, indicating that the adsorption does not occur uniformly across the adsorbent surface. The Freundlich isotherm equation is often employed to determine the adsorption capacity and the intensity of adsorption^85^. The linearized form of Langmuir and Freundlich isotherm is presented in Table 3.

**Fig. 7:**
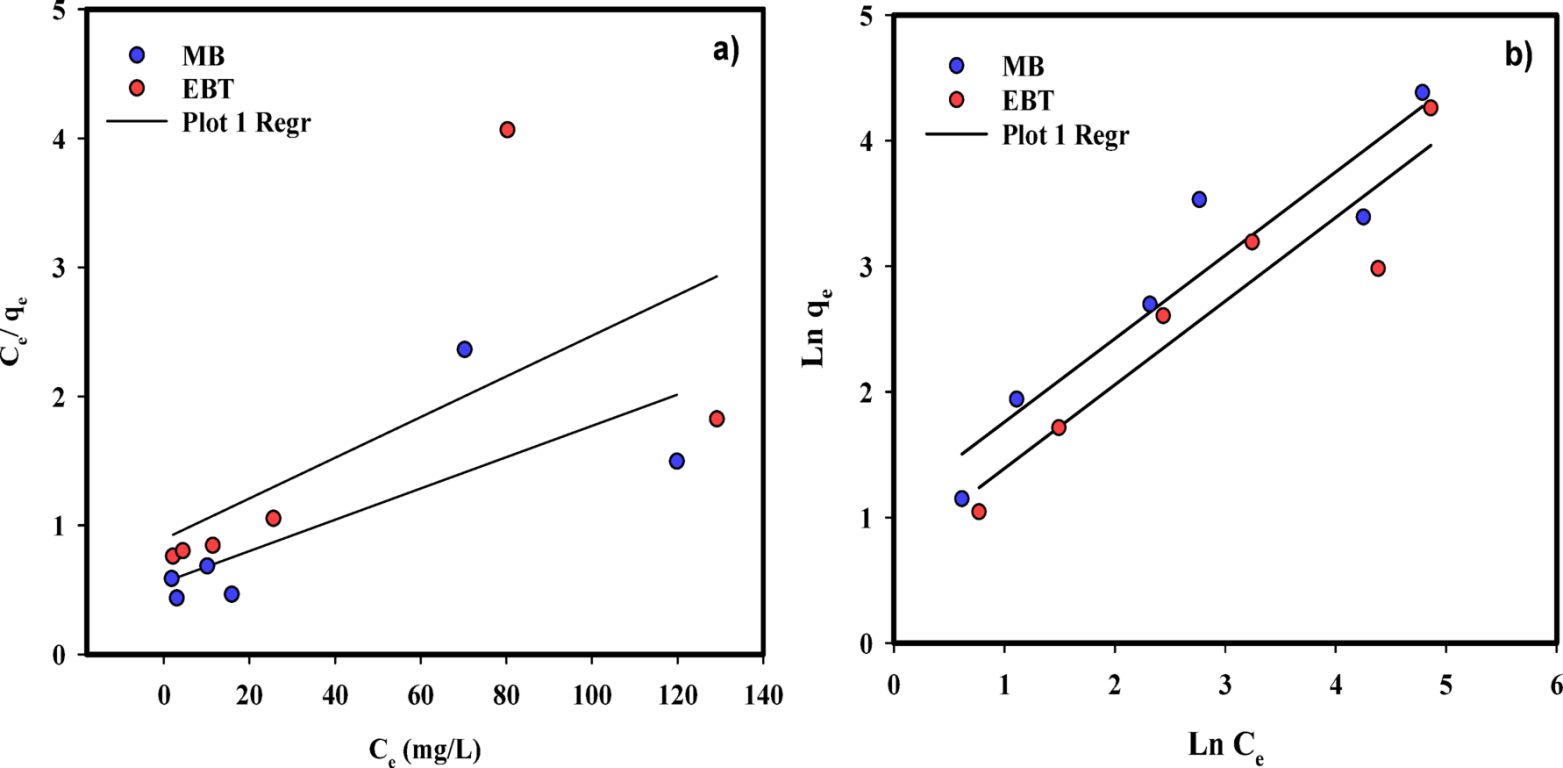
Adsorption isotherm of MB and EBT adsorption on NaAlg/BLAC/OMMT_3_ hydrogel, (**a**) Langmuir isotherm, and (**b**) Freundlich isotherm.

Table 4; Fig. 7a&b represent the determined values of the isotherm parameters for the removal of MB and EBT by NaAlg/BLAC/OMMT_3_ hydrogel at the initial concentration of dyes within a range of 5-100 mg/L, at a pH of 7, and 10 mg of the adsorbent for 24 h. The results obtained that the maximum adsorption capacity of MB and EBT removal by NaAlg/BLAC/OMMT_3_ hydrogel was found to be 136.98 and 66.66 mg/g, respectively. Also, the “n” values calculated by the Freundlich model are 1.508, and 1.501 for MB and EBT, respectively. Moreover, compared to the R^2^ value of Langmuir isotherm, the Freundlich isotherm showed a greater R^2^ value. Consequently, it has demonstrated that the Freundlich isotherm model accurately reflected the experimental results of MB and EBT uptake onto the NaAlg/BLAC/OMMT_3_ hydrogel surface.

**Table 3:** The adsorption isotherm and kinetics models.

Adsorption isotherm	Equation	Parameters
Langmuir	$$\:\raisebox{1ex}{${C}_{e}$}\!\left/\:\!\raisebox{-1ex}{${q}_{e}$}\right.=\raisebox{1ex}{${C}_{e}$}\!\left/\:\!\raisebox{-1ex}{${Q}_{m}$}\right.+\:\raisebox{1ex}{$1$}\!\left/\:\!\raisebox{-1ex}{$b{Q}_{m}$}\right.$$	C_e_ = the equilibrium concentration of dyes adsorbed in mg/L q_e_ = the amount of dyes adsorbed in mg/g Q_m_ = maximum adsorption capacity in mg/g b = the rate constant of Langmuir in L/mg
Freundlich	$$\:\text{ln}{q}_{e}\:=\text{ln}{K}_{f}-\raisebox{1ex}{$1$}\!\left/\:\!\raisebox{-1ex}{$n$}\right.\text{ln}{C}_{e}$$	C_e_ = the equilibrium concentration of dyes adsorbed in mg/L q_e_ = the amount of dyes adsorbed in mg/g K_f_ = the rate constants of Freundlich in L/mg n = the capability and strength of the uptake process
kinetics models	**Equation**	**Parameters**
pseudo-first-order	$\:\text{log}\left({q}_{e}-{q}_{t}\right)=\text{log}{q}_{e}-\frac{{K}_{1}}{2.303\:t}$	q_e_ = the amount of dyes adsorbed in mg/g q_t =_ the adsorption capacity (mg/ g) of the adsorbent at time t. k_1_ (1/h) = the pseudo-first-order rate constant
pseudo-second-order	$$\:\raisebox{1ex}{$t$}\!\left/\:\!\raisebox{-1ex}{${q}_{e}$}\right.=\:\raisebox{1ex}{$1$}\!\left/\:\!\raisebox{-1ex}{${K}_{2}$}\right.{q}_{e}^{2}+\:\raisebox{1ex}{$1$}\!\left/\:\!\raisebox{-1ex}{${q}_{e}$}\right.t$$	q_e_ = the amount of dyes adsorbed in mg/g k_2_ (g/mg/h) = the pseudo-second-order rate constant
Intraparticle diffusion model	$\:{q}_{t}={K}_{i}{t}^{0.5}+C$	qt = the adsorption capacity (mg/ g) of the adsorbent at time t. C= the intercept Ki = the intraparticle diffusion rate constant (mg/g min^0.5^)

**Table 4:** Parameters of Langmuir and Freundlich isotherms.

	Langmuir isotherm			Freundlich isotherm		
Adsorbate	Qm (mg/g)	b (L/mg)	R^2^	Kf (L/g)	n	R^2^
MB	136.98	0.021	0.570	1.126	1.508	0.884
EBT	66.66	0.017	0.396	1.125	1.501	0.887

### Adsorption kinetics

**Fig. 8:**
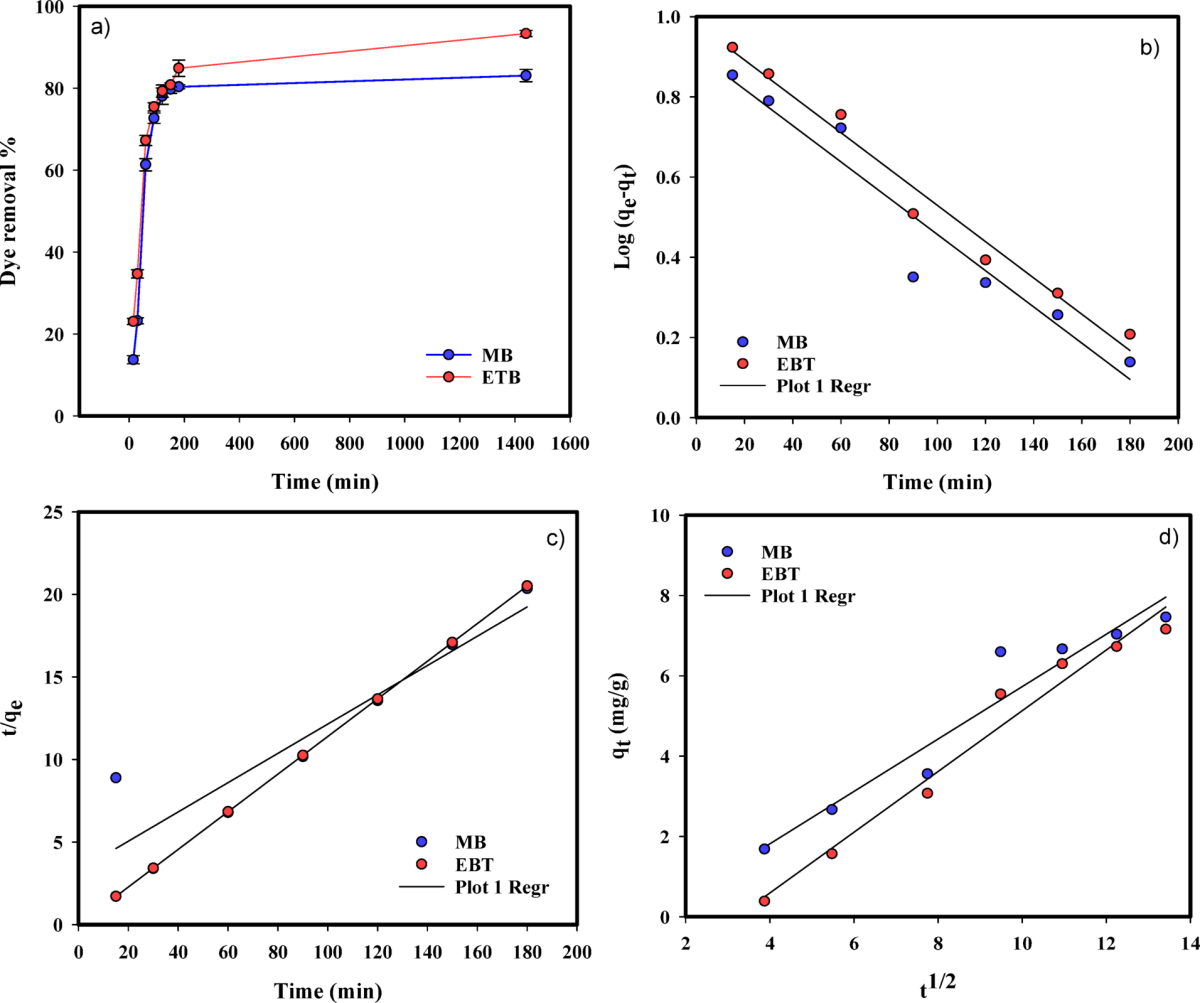
(**a**) Effect of time on removal of MB and EBT by NaAlg/BLAC/OMMT_3_ hydrogel, and Regressions of kinetic plots for the adsorption of MB and EBT adsorption on NaAlg/BLAC/OMMT_3_ hydrogel; (**b**) Pseudo-first-order modal, (**c**) Pseudo-second-order modal, and (**d**) intraparticle diffusion model.

The adsorption effectiveness of the NaAlg/BLAC/OMMT3 hydrogel was examined through the manipulation of contact time ( 15–180 min) and evaluated at equilibrium after 24 h, while maintaining other parameters constant; initial dye concentration of 10 mg/L, a pH value of 7, and 10 mg of the adsorbent. Figure 8a shows the increase in the dye removal percentage with increase in contact time can be attributed to the fact that more time will be available for dye to an attractive complex with hydrogel. For both Methylene blue (MB) and eriochrome black-T (EBT) dyes, the dye removal percentage increased with increasing initial contact time from 15 to 180 min. At contact times higher than 180 min for 24 h, the removal percentage did not change owing to the saturation of the active sites of the adsorbent. For that reason, the results illustrated that the maximum removal percent using the NaAlg/BLAC/OMMT_3_ hydrogel on removal of MB and EBT dyes was 80.3%, and 84.9%, respectively at 90 min. In the beginning time from 15 to 90 min, the faster rates of dye adsorption can be attributed to the abundance of binding sites available for adsorption on the hydrogel, After 90 min, from 120 to 180 nim the slower adsorption rates observed towards the end are a result of the saturation of the binding sites and the attainment of equilibrium^32,76,86,87^.

The rate at which a dye is absorbed and the effectiveness of the adsorption process on NaAlg/BLAC/OMMT_3_ hydrogel can be determined by studying the kinetics of adsorption. This analysis also helps identify potential applications for the adsorbent. The adsorption mechanism relies on both the chemical and physical properties of the adsorbent, as well as the mass transfer process^76,88^. Additionally, studying the kinetics of the adsorption process is crucial for designing effective adsorbents, as it provides valuable information about the underlying mechanisms and the rate at which pollutants are taken up^89,90^. The literature indicated that the process of removing pollutants can be effectively described by employing various kinetic models, including pseudo-first-order, pseudo second-order, and intraparticle diffusion models, illustrated in Table 3.

The linear plots of log(q_e_ - q_t_) versus t and (t/q_t_) versus t, and q_t_ versus t^0.5^ were calculated for the are drawn for the pseudo-first-order, the pseudo-second-order models, and intraparticle diffusion models, respectively (Fig. 8b-d). The values of k_1_ can be determined from the slope of the linear plot of log(q_e_ - q_t_) versus t, k_2_ can be calculated from the slope of the linear plot (t/q_t_) versus t, and K_i_ can be calculated from the slope of the linear plot q_t_ versus t^0.5^. The values of K_1_, K_2_, K_i_, q_e_, and the correlation coefficient (R^2^) obtained from the linear plots are shown in (Table 5).

Comparisons were made regarding the square of the correlation coefficients (R^2^ obtained from three kinetic models applied to MB and EBT adsorption onto NaAlg/BLAC/OMMT_3_ hydrogel, as demonstrated in Table 5. In the case of EBT, the adsorption process exhibited kinetic control, as evidenced by the strong correlation coefficients of the pseudo second-order model (R^2^ = 0.999), which was followed by the pseudo-first-order model (R^2^ = 0.978). These findings suggest that the adsorption of EBT onto the hydrogel is primarily governed by chemical interactions, with physical adsorption playing a secondary role^89^. Conversely, for MB adsorption, both the pseudo-first-order and intraparticle diffusion models were found to exert kinetic control, yielding correlation coefficients of R^2^ = 0.931. This observation implies a two-stage adsorption process occurring on and within the adsorbent modelwithin the adsorbent model.

**Table 5:** Pseudo-first-order, Pseudo-second-order, and **I**ntraparticle diffusion kinetics models for MB and EBT onto NaAlg/BLAC/OMMT3 hydrogel at initial dye concentration of 10 mg/ L.

Adsorbate	Pseudo-first-order		
	q_e_ (mg/g)	K_1_ (1/min)	R^2^
MB	8.839	2.093	0.931
EBT	8.774	2.261	0.978
	**Pseudo-second-order**		
	q_e_ (mg/g)	K_2_(g/mg min)	R^2^
MB	8.839	0.0104	0.852
EBT	8.774	0.0128	0.999
	**Intraparticle diffusion model**		
	K_i_ (mg/g min^0.5^)	C (mg/g)	R^2^
MB	-0.791	0.652	0.931
EBT	-2.432	0.756	0.969

### Regeneration (desorption) study

**Fig. 9:**
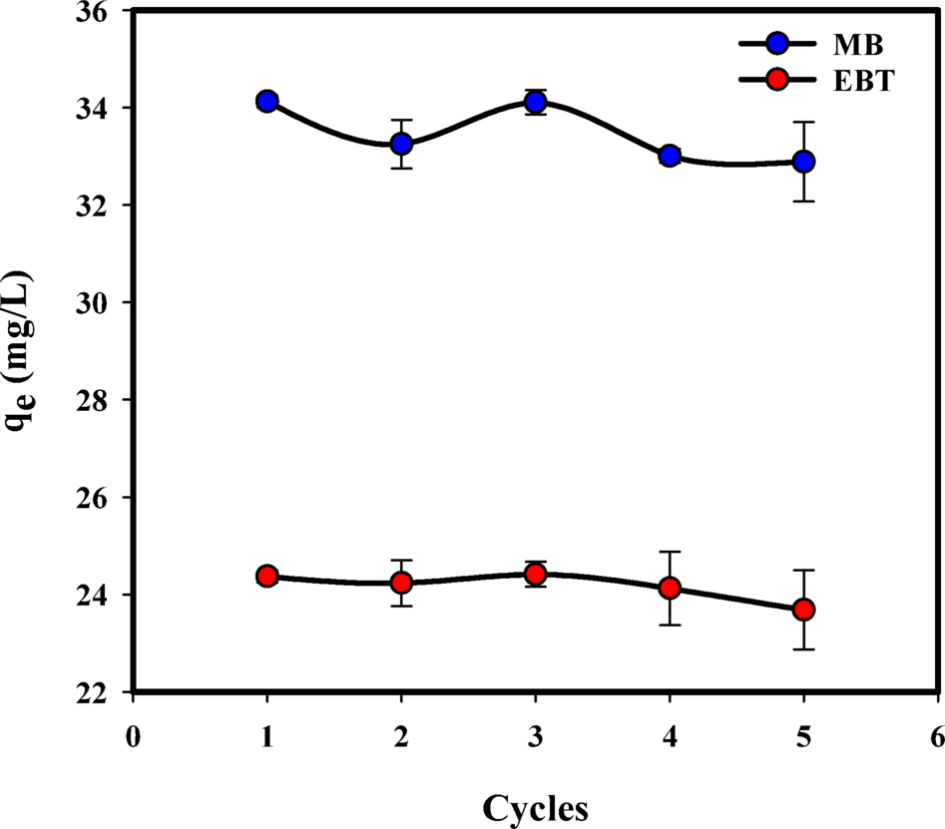
Desorption and regeneration experiment using NaAlg/BLAC/OMMT_3_ hydrogel for MB and EBT adsorption.

The regenerative property of an adsorbent proves highly beneficial for its potential applications. Desorption studies are conducted to assess the capacity of a used adsorbent to be recycled the dye once more. Therefore, the most effective adsorbent should possess a high adsorption capacity and exceptional regeneration (desorption) characteristics to ensure economic viability^94,95^. The regeneration efficiency of NaAlg/BLAC/OMMT_3_ hydrogel was investigated through five consecutive adsorption-desorption cycles in a 0.1 M HCl solution (pH 7), an initial dye concentration of 50 mg/L for 24 h at room temperature, as illustrated in Fig. 9. The results revealed that the adsorption capacity for MB and EBT dyes exhibited a very slight decrease after five cycles. The slight decrease in adsorption performance across cycles can be attributed to several factors, including partial pore blockage, reduced availability of active sites due to incomplete desorption, and possible structural changes in the hydrogel matrix after repeated use. This indicates that the NaAlg/BLAC/OMMT_3_ hydrogel can be utilized across multiple cycles, showcasing notable structural stability even with repeated use. The results obtained exhibited parallels with prior research on the regeneration of composite materials involving polyacrylic acid grafted carboxymethyl cellulose and activated carbon, for the removal of methylene blue (MB) dye [78]. Additionally, similarities were noted with studies on polyvinyl alcohol/porous carbon composite hydrogels for the removal of organic pollutants from wastewater [79], and with the utilization of hydrogel biocomposites (PPAC/TG) consisting of activated carbon derived from pomegranate peels (PPAC) embedded in tragacanth gum (TG) for the removal of crystal violet dye (CV) from aqueous solutions [80].

### Adsorption mechanism

**Fig. 10:**
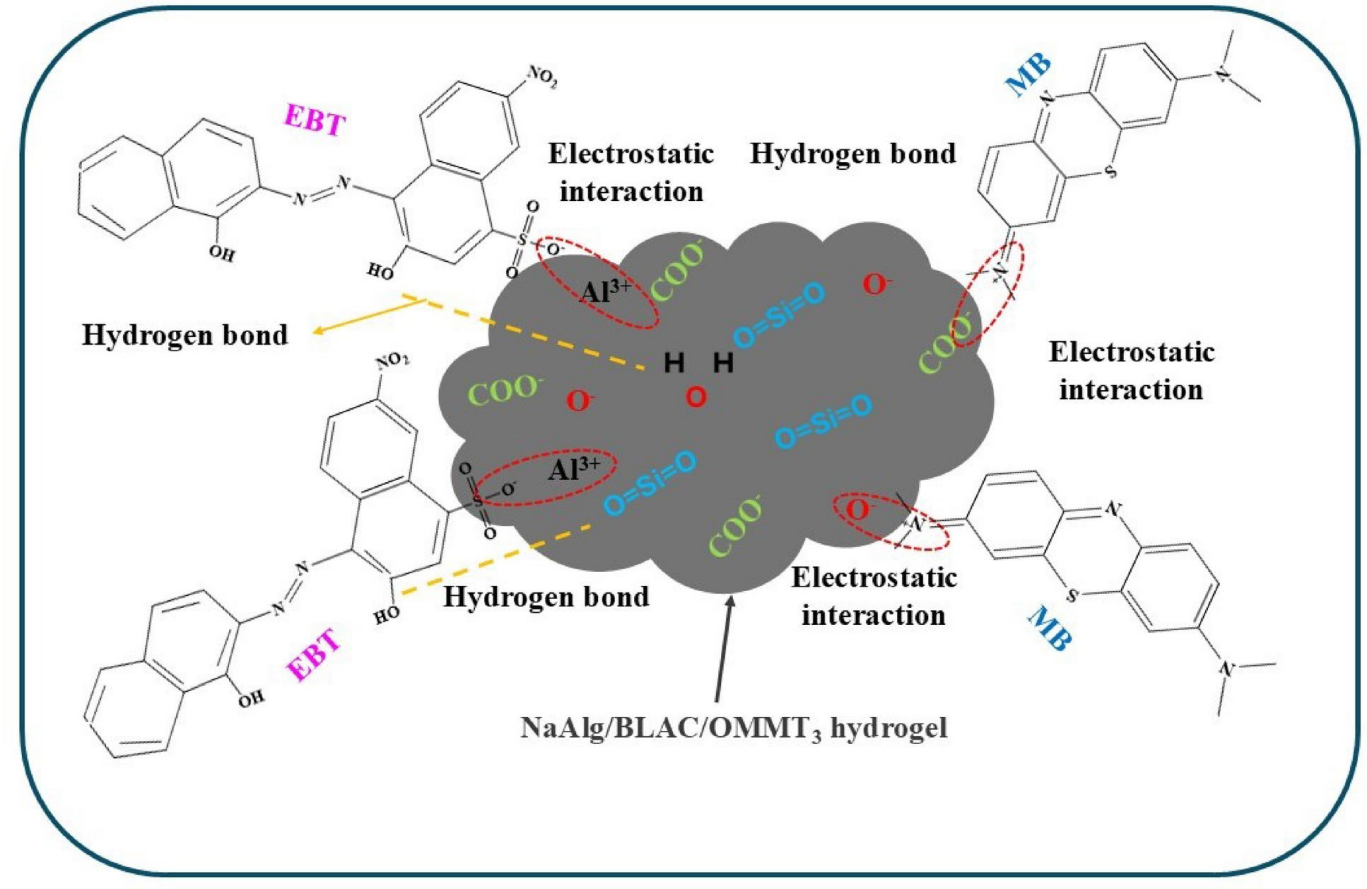
The proposed adsorption mechanism for MB and EBT onto NaAlg/BLAC/OMMT_3_ hydrogel.

**Fig. 11:**
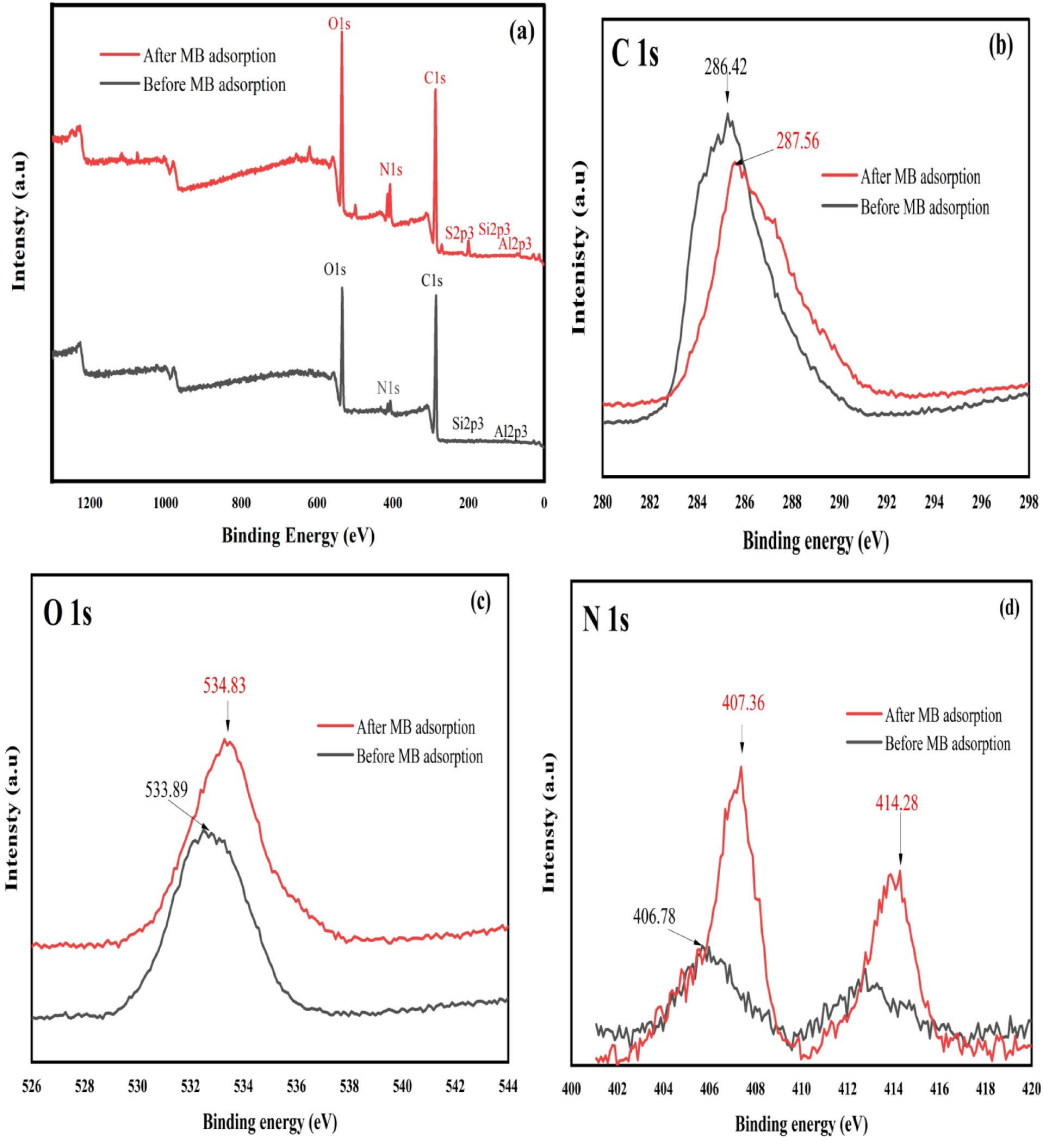
XPS of NaAlg/BLAC/OMMT_3_ hydrogel before and after MB adsorption.

The adsorption process of MB and EBT onto NaAlg/BLAC/OMMT_3_ hydrogel is shown in Fig. 10. The exceptional adsorption capacity of the NaAlg/BLAC/OMMT_3_ hydrogel for MB and EBT relies heavily on the internal folded structure of the active carbon and the modified montmorillonite clay, as well as the porosity of the hydrogel’s surface. FTIR and XPS analyses were performed to investigate the adsorption mechanism of MB and EBT onto the NaAlg/BLAC/OMMT_3_ hydrogel. The FTIR results, as discussed before (Fig. 1), revealed that electrostatic interactions between the cationic and anionic dyes and the hydrogel network likely caused the slight shifts in absorption peak frequencies. These changes in absorbance indicate the involvement of dye-binding mechanisms at the active sites of the adsorbent. XPS analysis provided further insights into MB adsorption. High-resolution XPS spectra of the hydrogel before and after MB adsorption (Fig. 11) revealed key changes in O 1s, N 1s, and C 1s peaks. Before adsorption, the C 1s and O 1s peaks appeared at ~ 286.4 eV and ~ 533.8 eV, respectively, corresponding to functional groups such as C = O/C-O, C–C, C–N, and C–O (Fig. 11b&c)^96,97^. After MB adsorption, these peaks shifted to ~ 287.5 eV for C 1s and ~ 534.8 eV for O 1s, confirming the successful adsorption of MB onto the hydrogel^98^. Additionally, a new peak at ~ 169.9 eV (S 2p) emerged after adsorption, indicating the presence of sulfur species from MB. The significant shift in the C = O/C-O peak implies complex formation between MB and hydrogel oxygen-containing groups (O-MB)^99^, while the increase in C 1s binding energies suggests interactions between MB and adjacent nitrogen or oxygen atoms^100^. The N 1s peak in the hydrogel before adsorption, attributed to the quaternary ammonium groups of MMT modified with cetyltrimethylammonium bromide, appeared at ~ 406.7 eV. After MB adsorption, this peak shifted to ~ 407.3 eV, alongside the emergence of a new peak at ~ 414.8 eV Fig. 11d. MB contains nitrogen atoms in its aromatic rings (thiazine group) and + N(CH₃)₂ groups. The appearance of the ~ 414.8 eV peak strongly indicates a chemical interaction or reaction between MB and the hydrogel composite, attributed to nitrogen KLL Auger electron emission from the MB molecule^101,102^. This suggests notable changes in the electronic environment of the nitrogen atoms in MB’s thiazine structure. The adsorption mechanism involves multiple interactions: (i) Electrostatic attraction occurs between the positively charged nitrogen in MB and the negatively charged montmorillonite clay and sodium alginate. Conversely, the anionic dye EBT interacts electrostatically with the positively charged functional groups in the hydrogel, such as quaternary ammonium groups from the modified clay. These interactions facilitate rapid adsorption by promoting strong attraction between oppositely charged species, thereby enhancing the hydrogel’s selectivity for both cationic and anionic dyes. (ii) π–π stacking interactions take place between the aromatic rings of MB and EBT, contributing to higher adsorption capacity by enabling dye molecules to align with the carbon-rich adsorbent surface, including activated carbon. (iii) Potential hydrogen bonding and hydrophobic interactions are facilitated by NaAlg and OMMT, further supporting the adsorption process^103,104^. This observation highlights the strong affinity and interaction mechanisms between MB and EBT and the hydrogel composite, confirming its potential as an effective adsorbent for dye removal in wastewater treatment applications.

On other hand, Table 6 presents a comparison of the adsorption capacity of the NaAlg/BLAC/OMMT3 hydrogel composite with other hydrogel composites previously utilized for the removal of MB and EBT dyes. Notably, studies on hydrogel composites for EBT removal are scarce in the literature. As indicated, the NaAlg/BLAC/OMMT_3_ hydrogel demonstrates significant potential as a promising material for the removal of both cationic and anionic dyes. It achieves high maximum adsorption efficiencies for MB and EBT dyes, reaching up to 80.3% and 84.9%, respectively, within a contact time of 90 min at pH 7.

**Table 6:** Comparison of the adsorption capacity of NaAlg/BLAC/OMMT3 hydrogel composite with other hydrogel composites used for the removal of MB and EBT.

Adsorbents	Dyes	Adsorption capacity (mg/g)	Reference
Nanocellulose on sodium alginate/polyacrylamide hydrogel (SA/PAAm/TCNFs)	MB	51	^105^
Poly(vinyl alcohol)-sodium alginate-chitosan-montmorillonite nanosheets (MMTNS)	MB	137.2	^106^
Carboxymethyl cellulose/activated carbon/hydroxyapatite (CMC/AC/HAp)	MB	43.86	^90^
Chitosan–montmorillonite/polyaniline	MB	111	^107^
Chitosan/GO composite adsorbent	MB	81.5	^108^
NaAlg/BLAC/OMMT_3_	MB	136.98	This study
Alginate based Bio-composite of basil seed mucilage	EBT	2.8	^52^
(Dihydroxypropyl) chitosan/glycidyl methacrylate-g-acrylamide (DHPC-GMA-g-Aam)	EBT	38.02	^109^
NaAlg/BLAC/OMMT_3_	EBT	66.66	This study

### Cytotoxicity of NaAlg/BLAC/OMMT_3_ hydrogel and hydrogel after adsorption MB and EBT against Vero and WRL-68 cell line

**Fig. 12:**
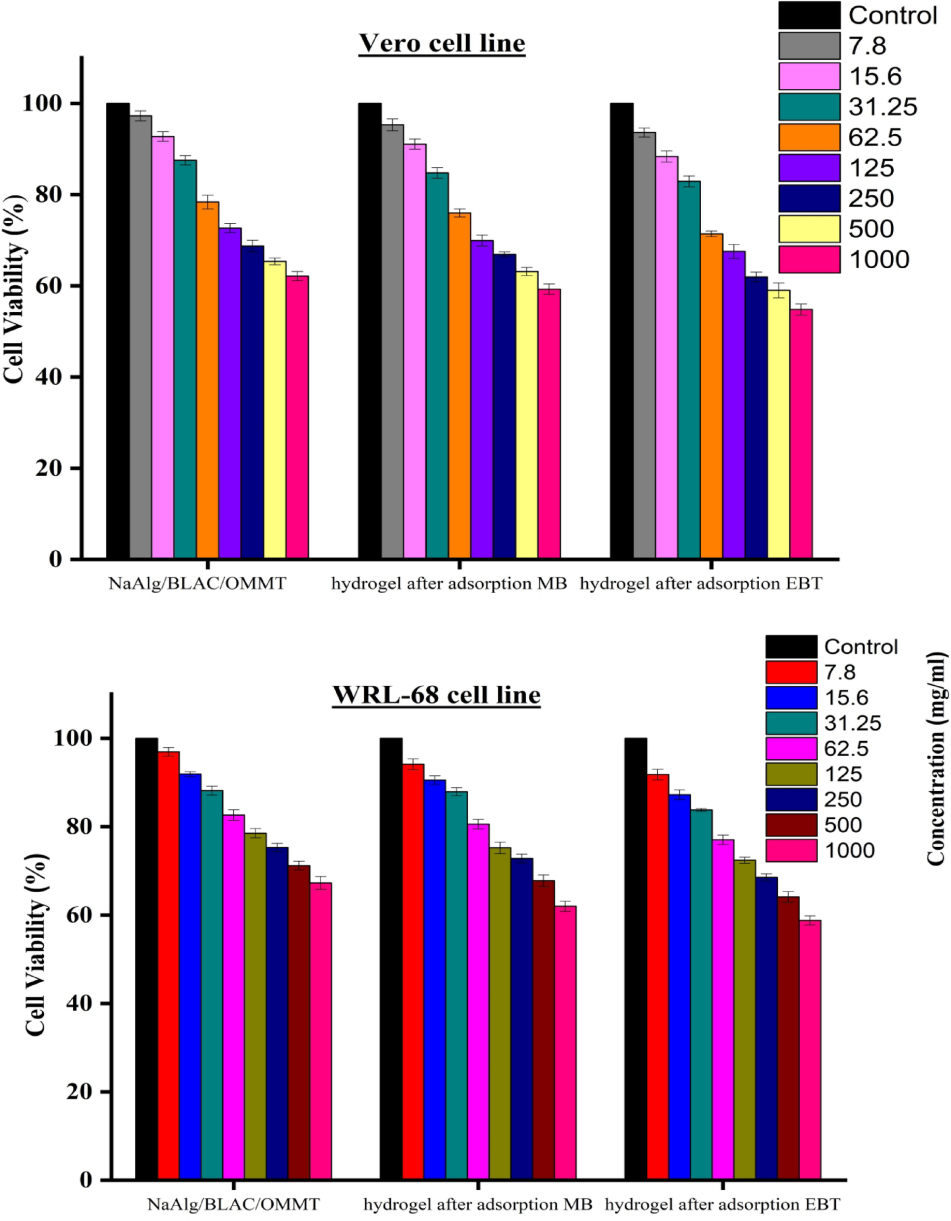
Cell viability (%) of NaAlg/BLAC/OMMT_3_ hydrogel and hydrogel after adsorption MB and EBT against Vero and WRL-68 cells at different concentrations after 24 h (*n* = 3) ± SD.

The potential cytotoxic effects of NaAlg/BLAC/OMMT_3_ hydrogel and hydrogel after adsorption MB and EBT against on Vero and WRL-68 cells were assessed using the MTT assay, as depicted in Fig. 12. The cells were treated with various concentrations of these compounds and incubated for 24 h. Control cells received only the diluent. The results indicate that Both cell lines (WRL-68 and Vero) show a clear dose-dependent decrease in cell viability as the concentration of the hydrogel increases from 7.8 µg/ml to 1000 µg/ml across all treatment conditions. NaAlg/BLAC/OMMT_3_ hydrogel shows the least cytotoxicity among the three treatments followed by hydrogel after adsorption MB and after adsorption EBT. The cell viability of NaAlg/BLAC/OMMT_3_ decreases with increasing concentration. At the highest concentration (1000 µg/mL), cell viability is around 60% against WRL-68 cells and 65% on Vero cells.For hydrogel after Adsorption of MB, the cell viability at the same concentration was 55% and 60% against WRL-68 cells and vero cells, respectily. The most significant decrease in cell viability, with around 55% and 50% viability againist WRL-68 cells and vero cells for hydrogel after Adsorption of EBT. Up to 31.25 µg/ml, all treatments maintain relatively high cell viability (> 80%) for both cell lines.Significant decreases in cell viability are observed at concentrations of 62.5 µg/ml and above. The WRL-68 cell line appears slightly more sensitive to the treatments compared to the Vero cell line, especially at higher concentrations. The cytotoxicity assessment indicates that while the NaAlg/BLAC/OMMT_3_ hydrogel and its variants after adsorption of MB and EBT are generally biocompatible at lower concentrations, higher concentrations (especially of the EBT-adsorbed hydrogel) pose significant cytotoxic risks. These findings are crucial for determining safe application concentrations in water treatment processes, ensuring that the hydrogels do not adversely affect human health or the environment.

### Environmental sustainability and economic feasibility of sodium alginate-activated carbon/OMMT hydrogels

The evaluation of the environmental sustainability and economic viability of sodium alginate-activated carbon/OMMT hydrogels necessitates a comprehensive cost feasibility analysis to assess the practicality and performance of the synthesized materials. Cost-effective methodologies are pivotal for real-world applications, particularly in the domain of environmental remediation. The sodium alginate-activated carbon/OMMT hydrogels, which are both biodegradable and renewable, serve as a fundamental material in the synthesis, thereby enhancing the environmental sustainability of the entire process.

In accordance with the method outlined by Srinivasakannan and Bakar, activated carbon is derived from banana leaves, with each kilogram of banana leaves yielding approximately 5 gm of activated carbon. The production of activated carbon entails the use of sodium hydroxide (costing $0.013) as well as the energy expenditure associated with equipment such as a muffle furnace and a dryer, which incur costs of $2.03 and $1.88, respectively. Consequently, the production cost for one gram of activated carbon amounts to $0.784.

As detailed in Table 7, the cost estimation for the preparation of one gram of sodium alginate-activated carbon/OMMT hydrogel is approximately $0.279, which demonstrates a cost-effective alternative compared to previous studies^110-112^. In practical applications, 0.02 gm of hydrogel is utilized to remove dye from 25 mL of synthetic dye solution. To treat approximately one cubic meter of wastewater, 800 g of hydrogel would be required, incurring a cost of $235.40.

This cost analysis highlights the economic feasibility of the production process, while ensuring alignment with sustainable material development principles.

**Table 7:** Cost Estimation to prepare 10 g of each sodium alginate - activated Carbon/OMMT hydrogels (NaAlg/ BLAC/OMMT).

Chemicals	Amount	Cost in USD
Sodium alginate	2 gm	≈ 0.320 $
Activated carbon	0.25 gm	≈ 0.196 $
MMT	0.25 gm	≈ 0.021$
CTAB	0.0825 gm	≈ 0.041 $
CaCl_2_	0. 1 gm	≈ 0.010 $
Total yield of hydrogel	2 gm	≈ 0.588 $
Total cost for one gm ≈ 0.294 $		
Total cost for 10 gm ≈ 2.942 $		

## Conclusion

The present study successfully demonstrated the preparation of sodium alginate-based hydrogels incorporating activated carbon derived from banana leaves and organo-modified montmorillonite (NaAlg/BLAC/OMMT). The activated carbon was effectively produced from the abundant and renewable banana leaf biomass, and the montmorillonite clay was surface-modified using a cationic surfactant. The hydrogel synthesis using calcium chloride as the cross-linking agent resulted in the successful integration of the activated carbon and organo-modified montmorillonite within the sodium alginate matrix, as evidenced by the comprehensive characterization techniques.

The adsorption studies revealed the pH-dependent performance of the NaAlg/BLAC/OMMT hydrogels. The hydrogels also demonstrated promising removal capacities concerning the initial adsorbate concentration and contact time. The results obtained that the maximum adsorption capacity of MB and EBT removal by NaAlg/BLAC/OMMT_3_ hydrogel was found to be 136.98 and 66.66 mg/g, respectively at a pH of 7. Importantly, the cytotoxicity analysis reveals that all hydrogels maintain high cell viability at concentrations up to 31.25 µg/ml but show significant effects at higher levels. WRL-68 cells are slightly more sensitive than Vero cells. The EBT-adsorbed hydrogel is the most cytotoxic, emphasizing the need to optimize concentrations for safe use in water treatment to avoid adverse health and environmental impacts.

Given these findings, further research should focus on optimizing hydrogel dosages, improving biocompatibility through surface modifications, and implementing protective handling measures to enhance safety in practical applications. Additionally, the hydrogel’s performance should be validated under real wastewater conditions, considering the presence of competing contaminants, fluctuating pH, and complex organic and inorganic compositions. Future studies should also explore long-term stability, regeneration efficiency beyond five cycles, and large-scale applicability in continuous-flow systems to ensure its feasibility for industrial wastewater treatment.

The successful development of this multifunctional hydrogel highlights its potential as an effective, sustainable, and eco-friendly adsorbent for dye removal. With further optimizations and field-scale evaluations, it can serve as a viable solution for wastewater treatment and other environmental applications.

## Data Availability

All data generated or analysed during this study are included in this published article.
